# Supplementary material for: Dissection of the interaction between the intrinsically disordered YAP protein and the transcription factor TEAD
Source: eLife. 2017 Apr 21;6:e25068. doi: 10.7554/eLife.25068 (PMC5400505; doi:10.7554/eLife.25068)

Figure 4-source data 1 – hYAP Leu65 – α-helix pocket

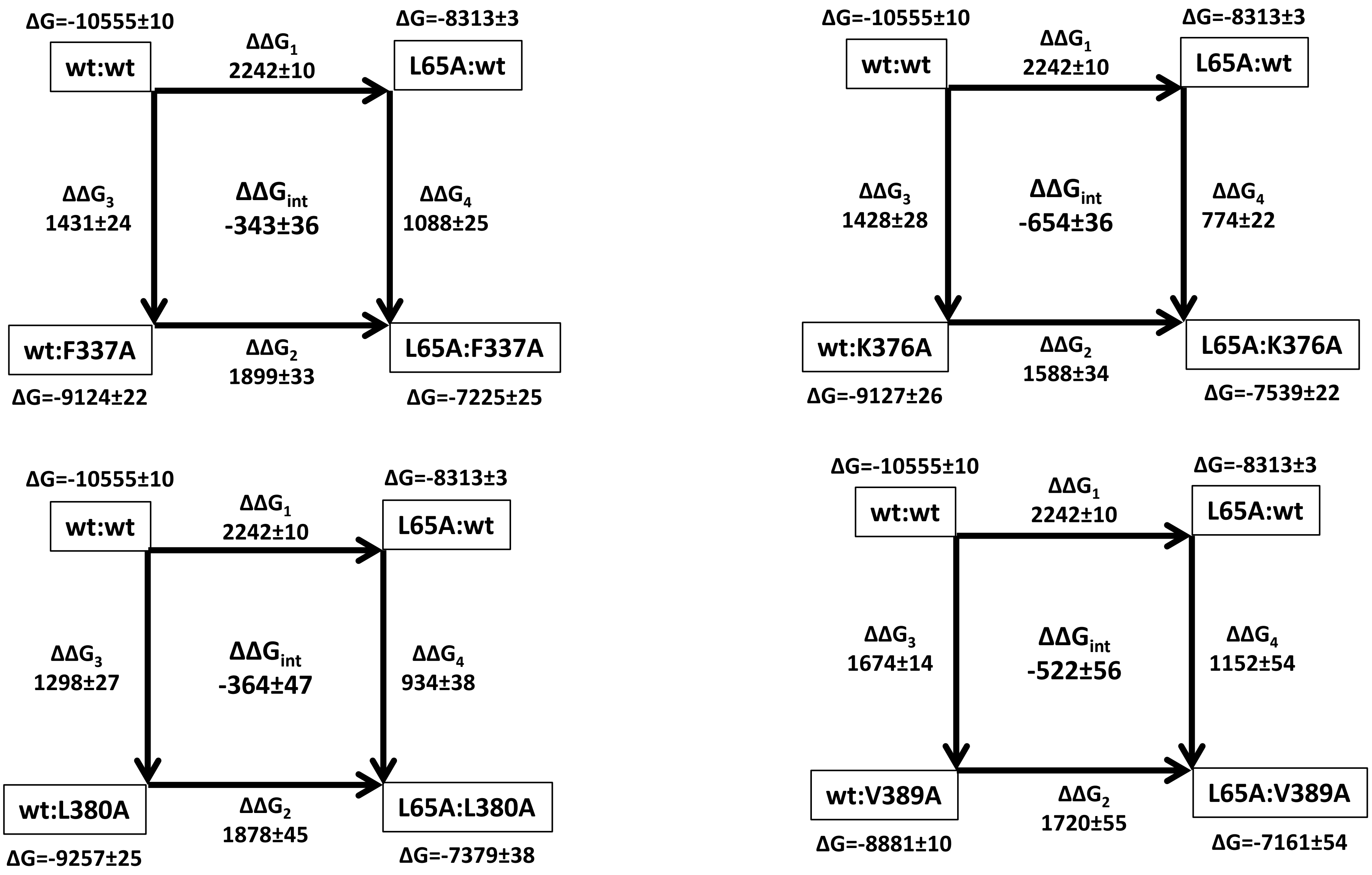

Figure 4-source data 1 – hYAP Leu65 – Ω-loop pocket

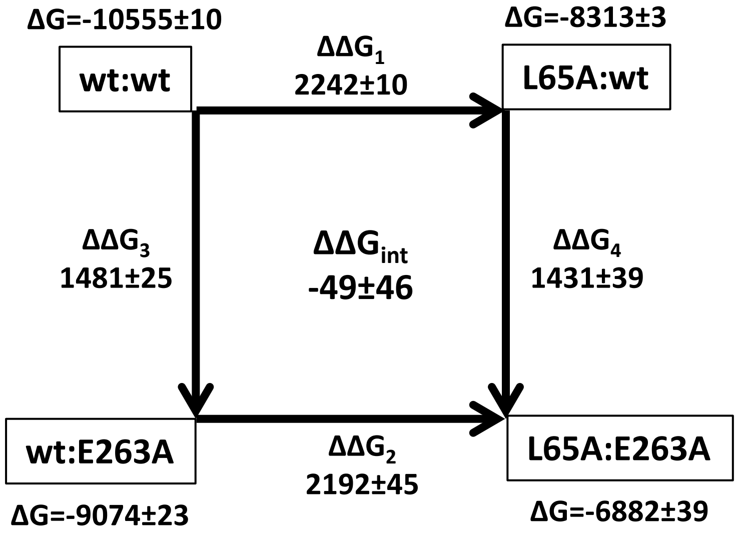

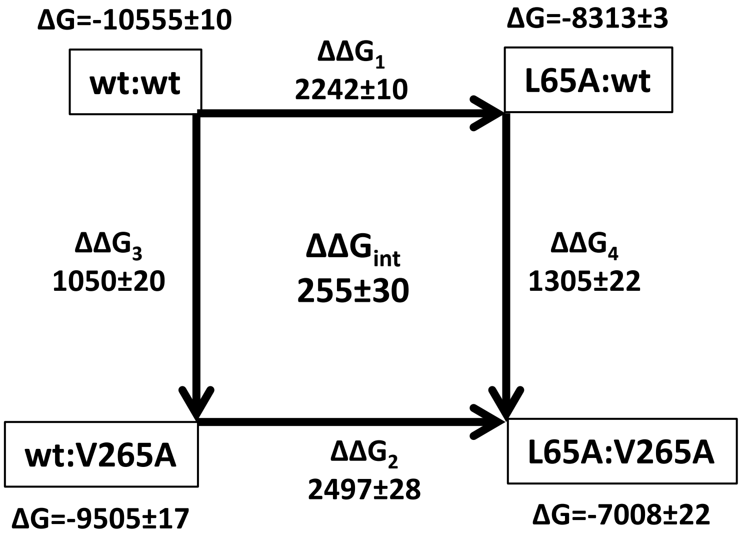

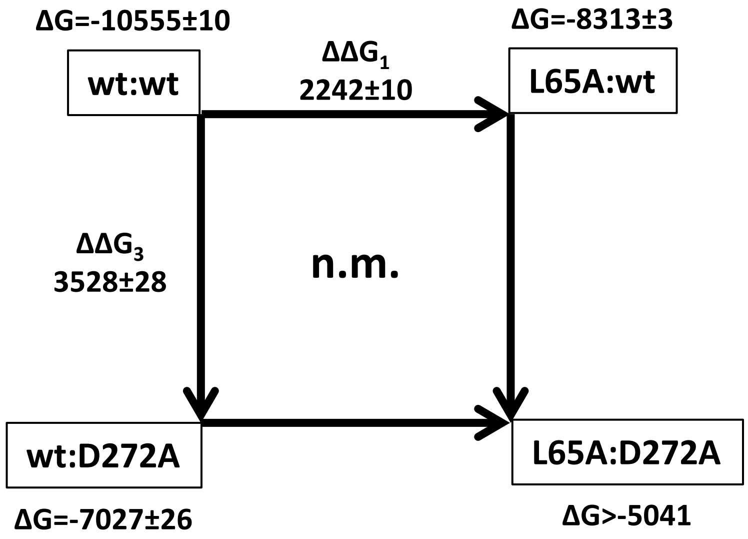

Figure 4-source data 1 – hYAP Leu68 – α-helix pocket

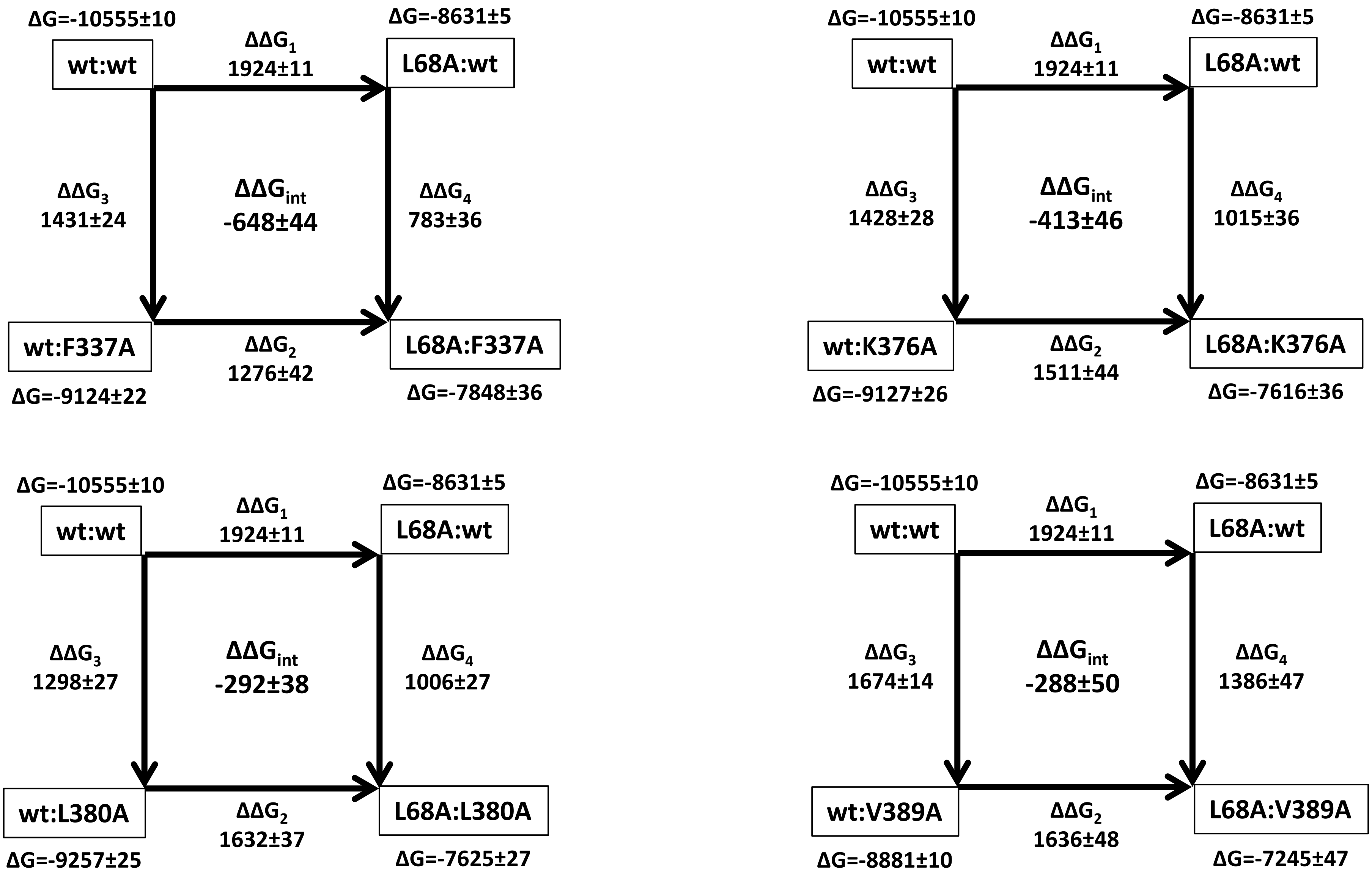

Figure 4-source data 1 – hYAP Leu68 – Ω-loop pocket

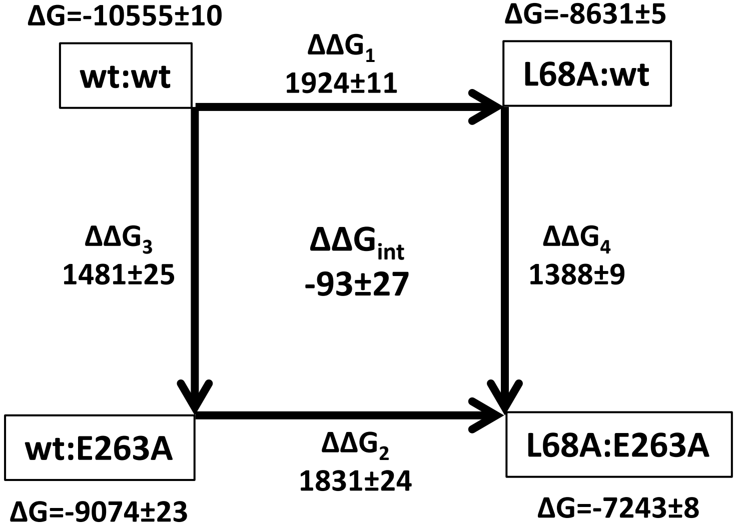

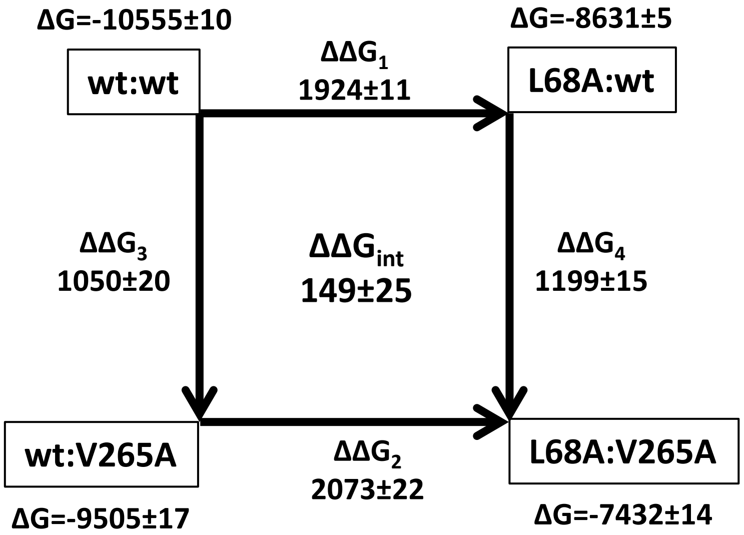

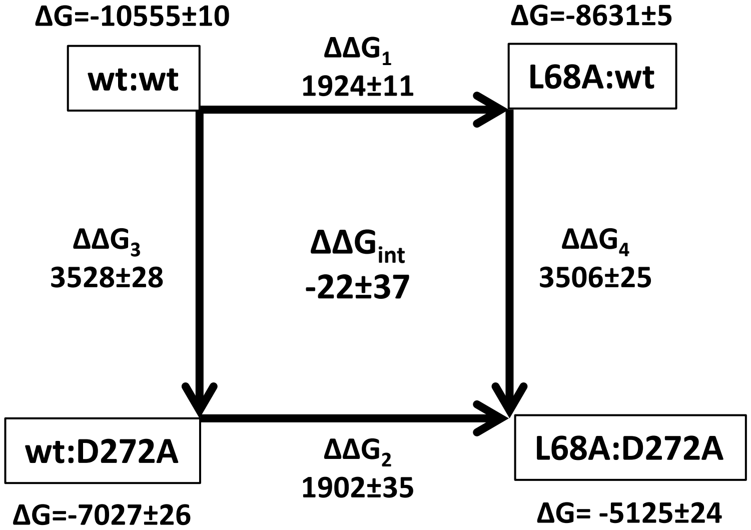

Figure 4-source data 1 – hYAP Phe69 – α-helix pocket

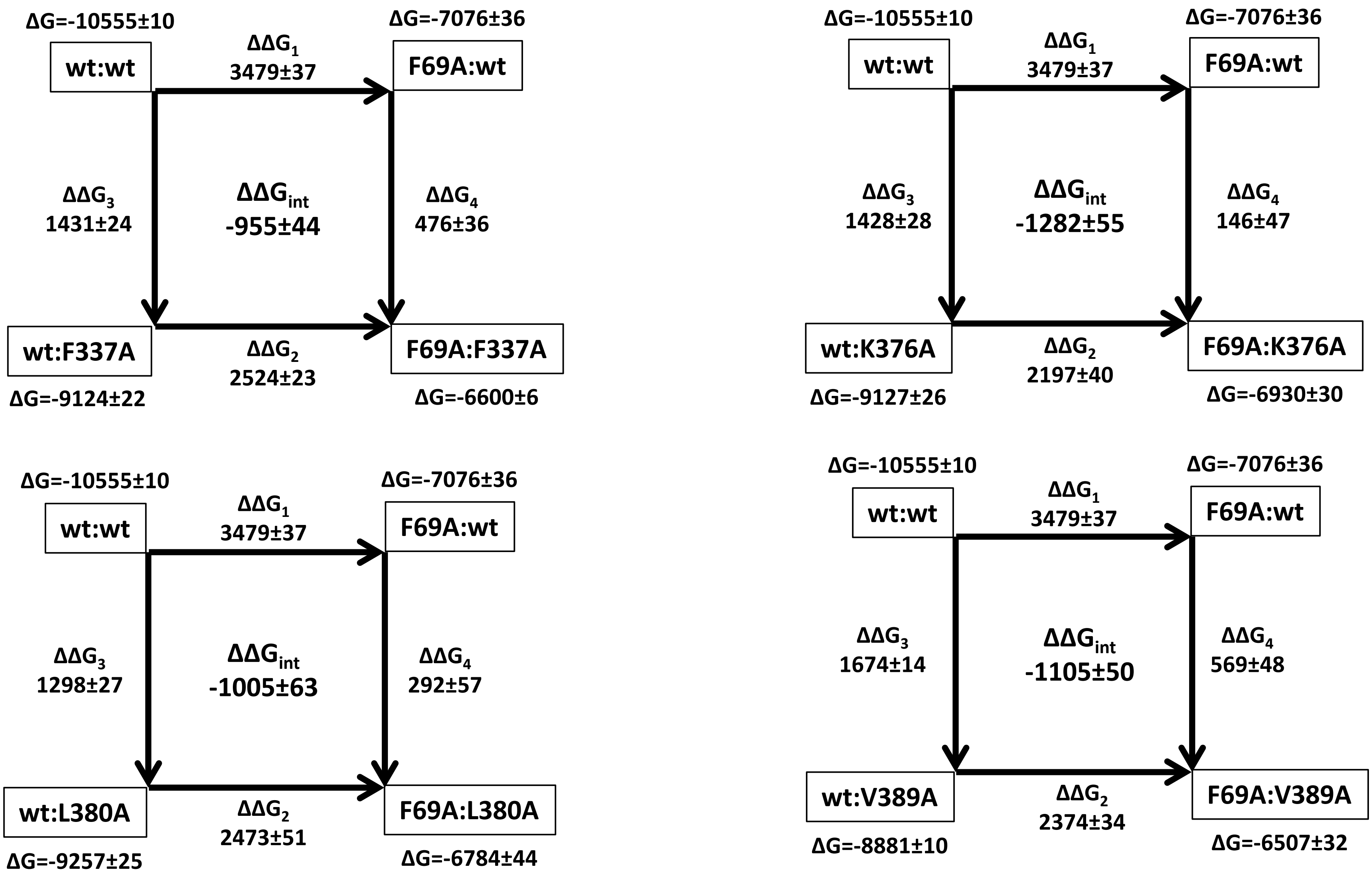

Figure 4-source data 1 – hYAP Phe69 – Ω-loop pocket

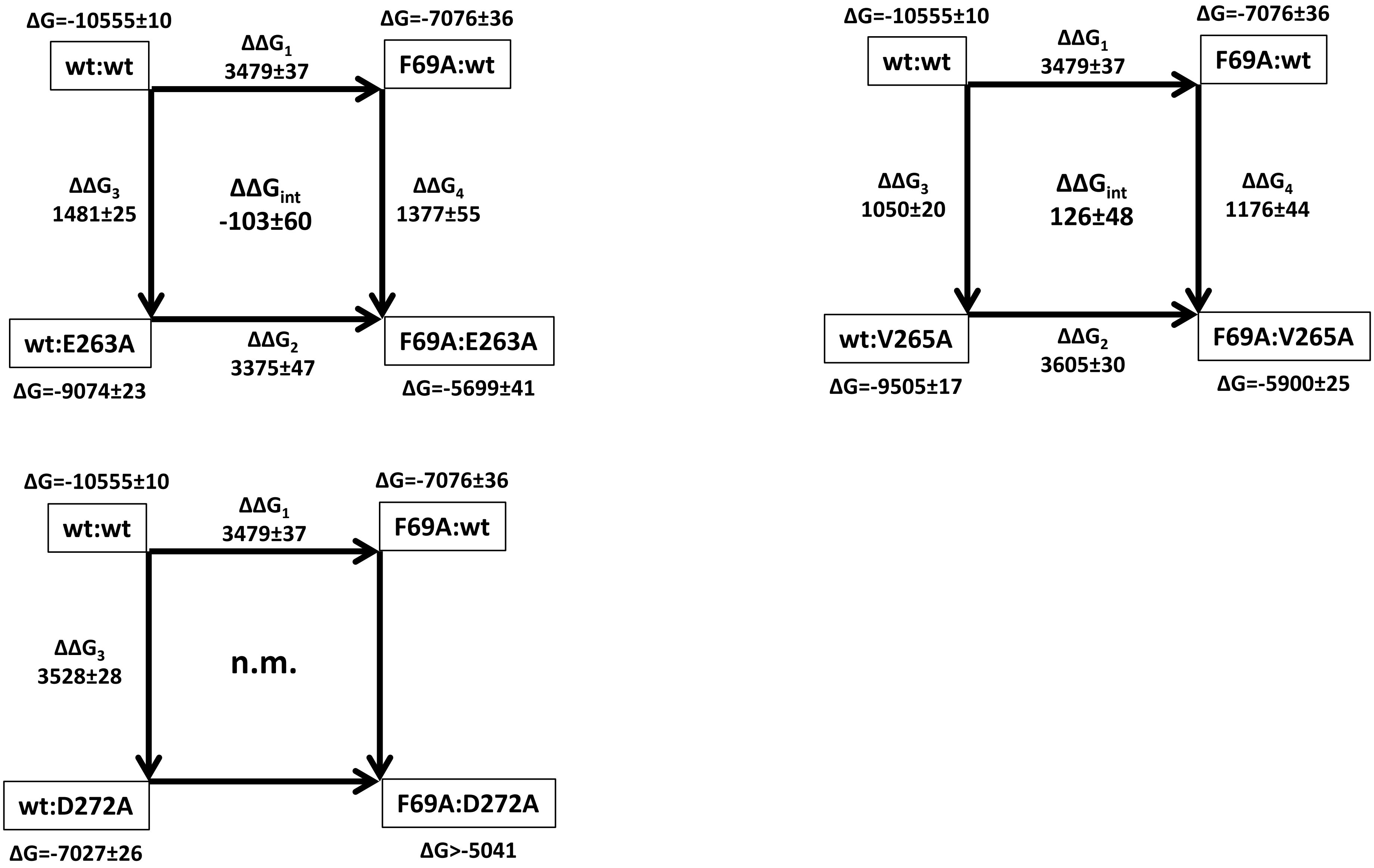

Figure 4-source data 1 – hYAP Met86 – α-helix pocket

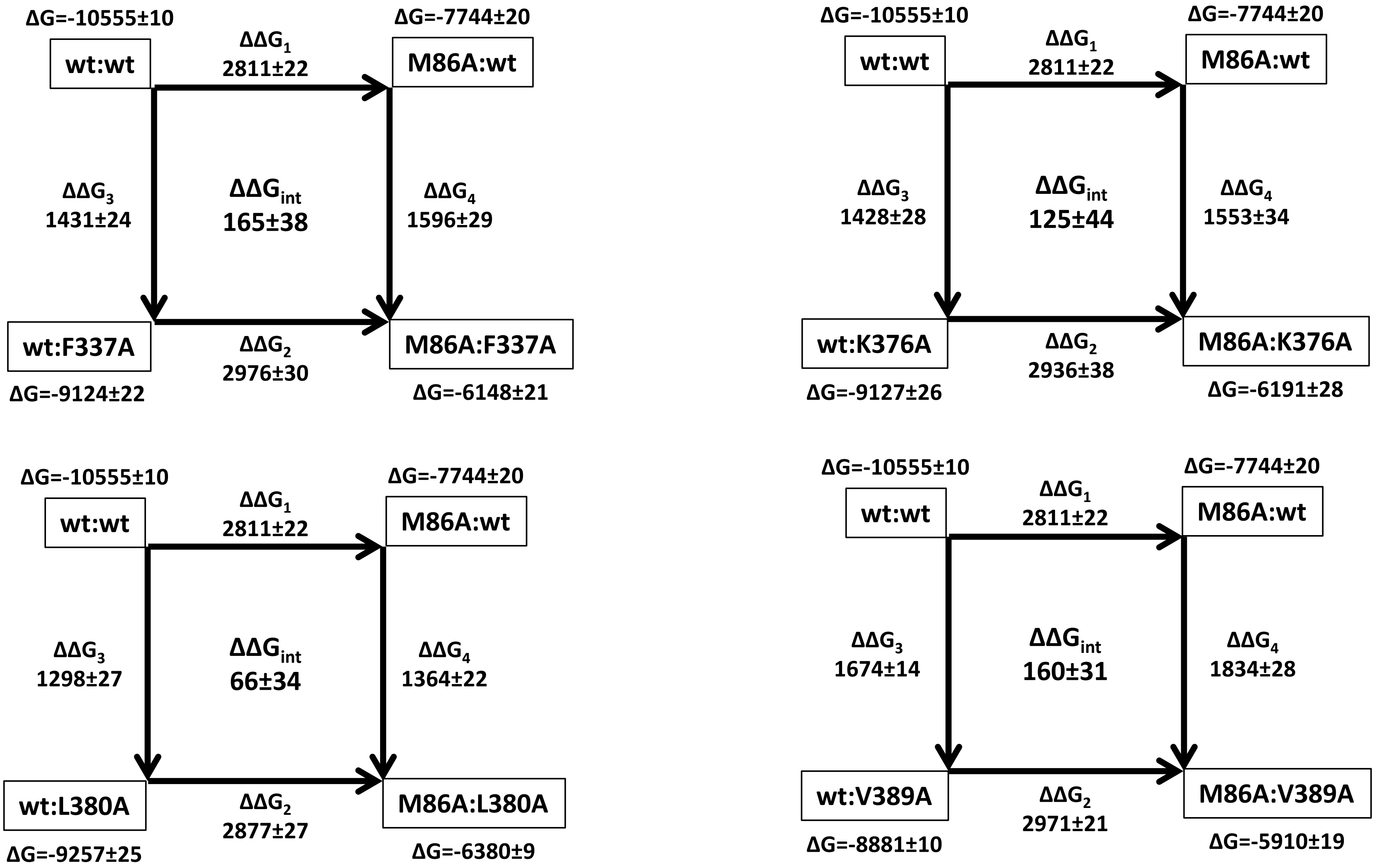

Figure 4-source data 1 – hYAP Met86 – Ω-loop pocket

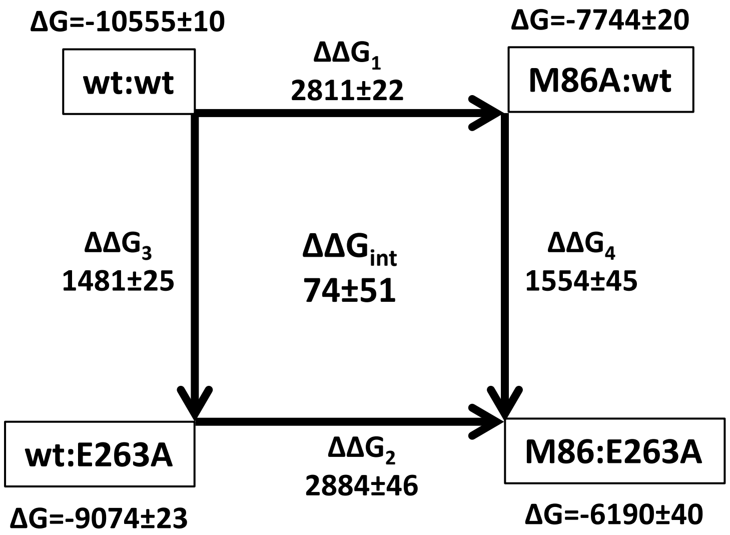

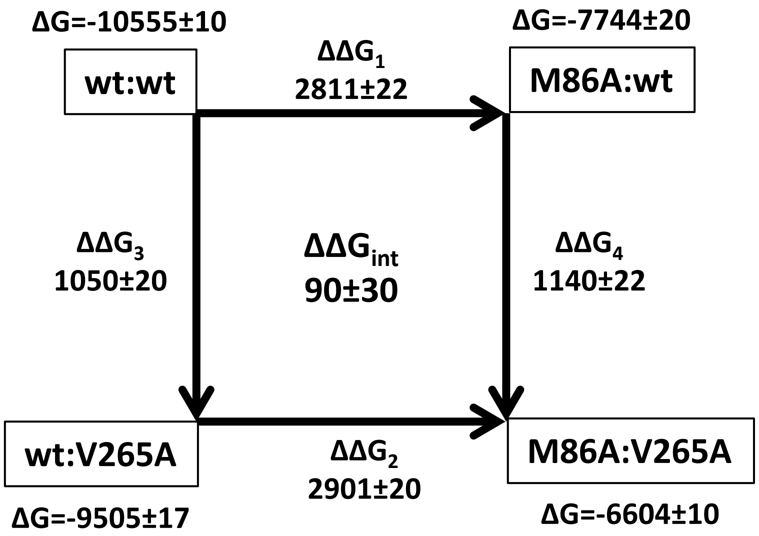

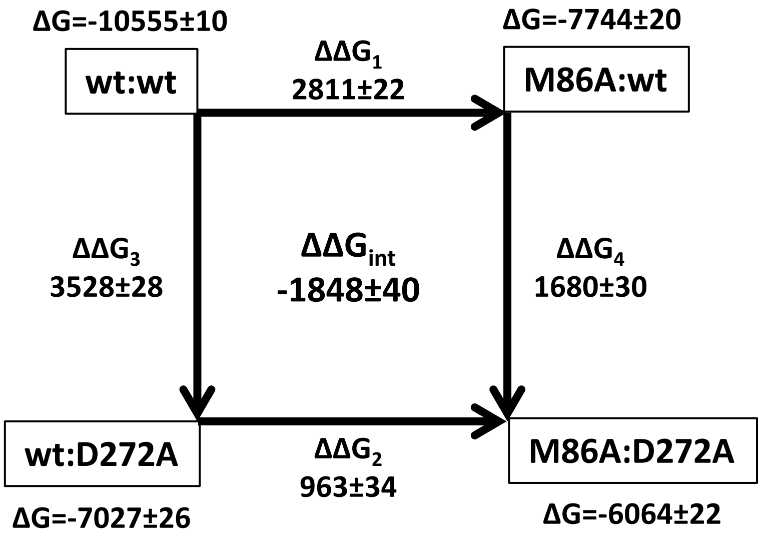

Figure 4-source data 1 – hYAP Arg89 – α-helix pocket

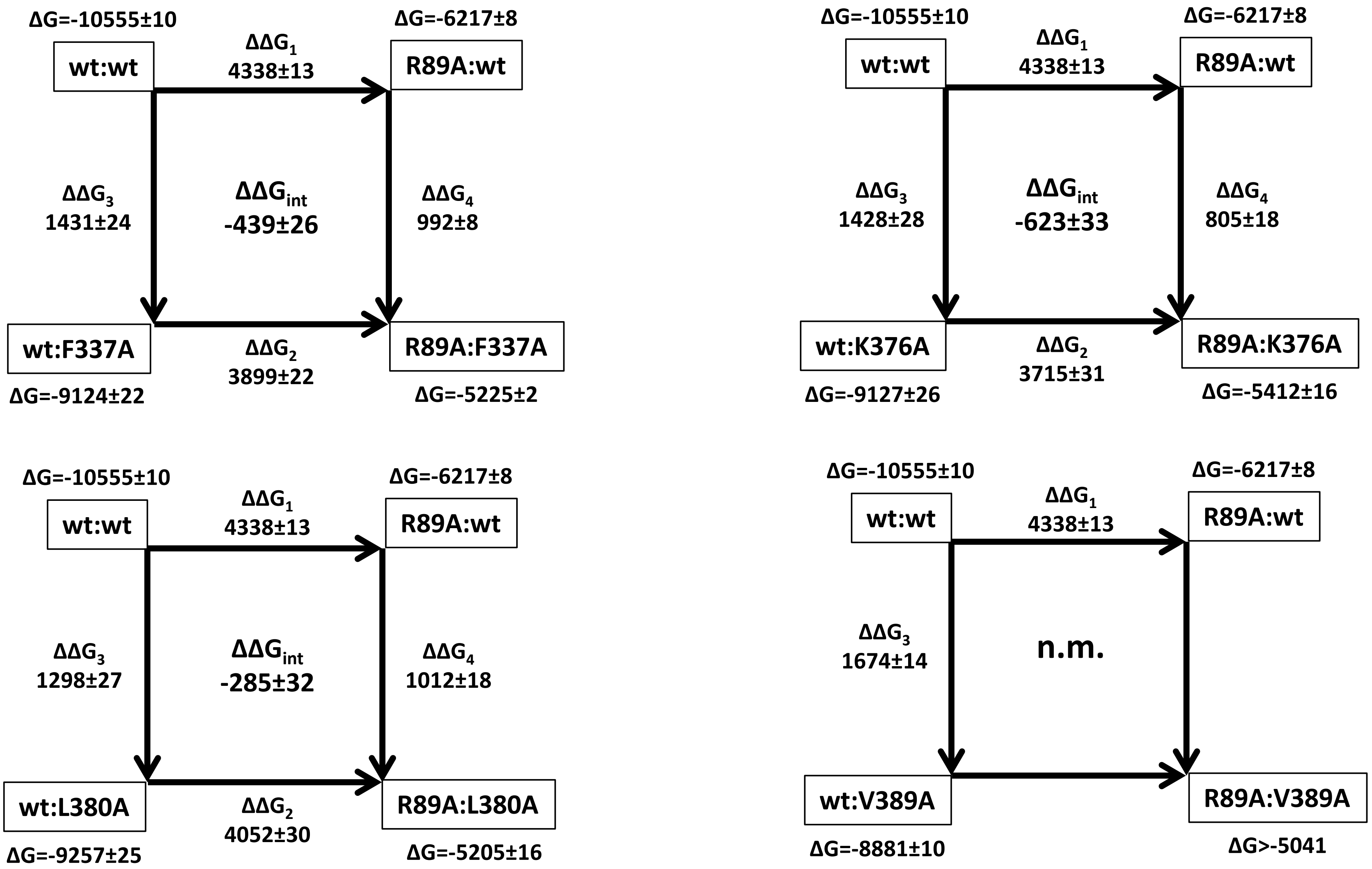

Figure 4-source data 1 – hYAP Arg89 – Ω-loop pocket

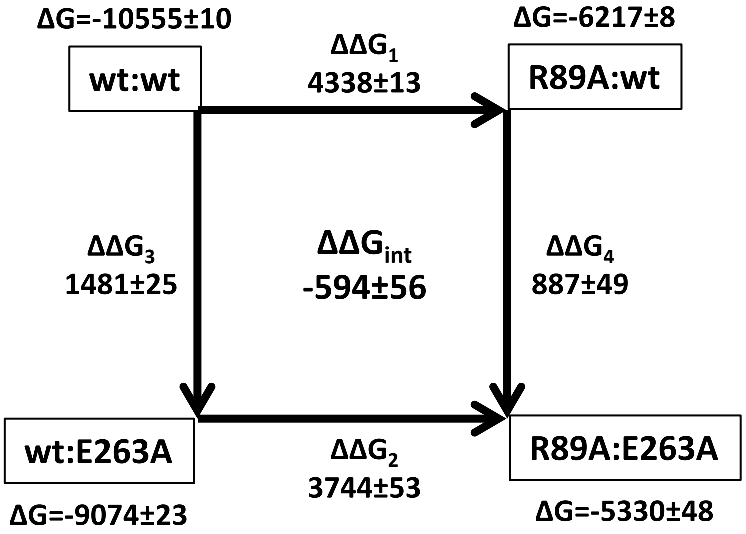

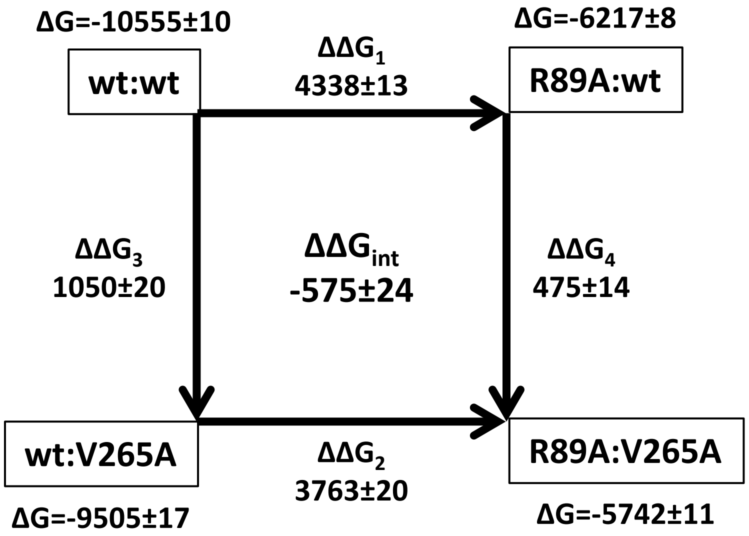

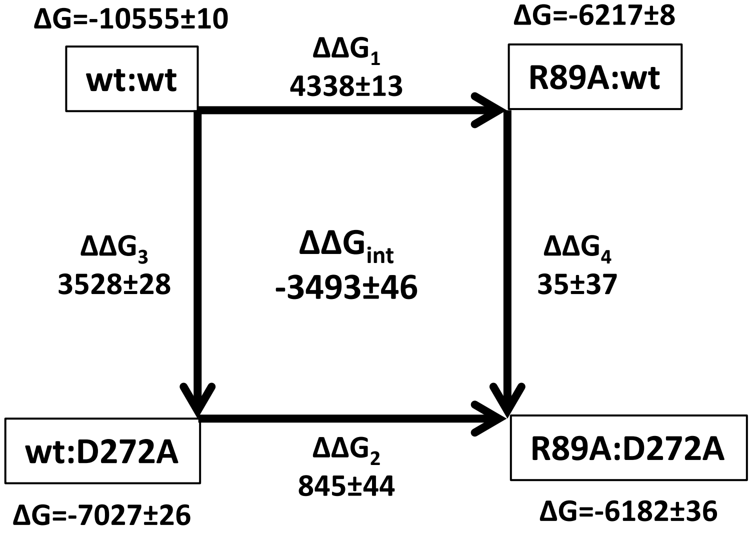

Figure 4-source data 1 – hYAP Leu91 – α-helix pocket

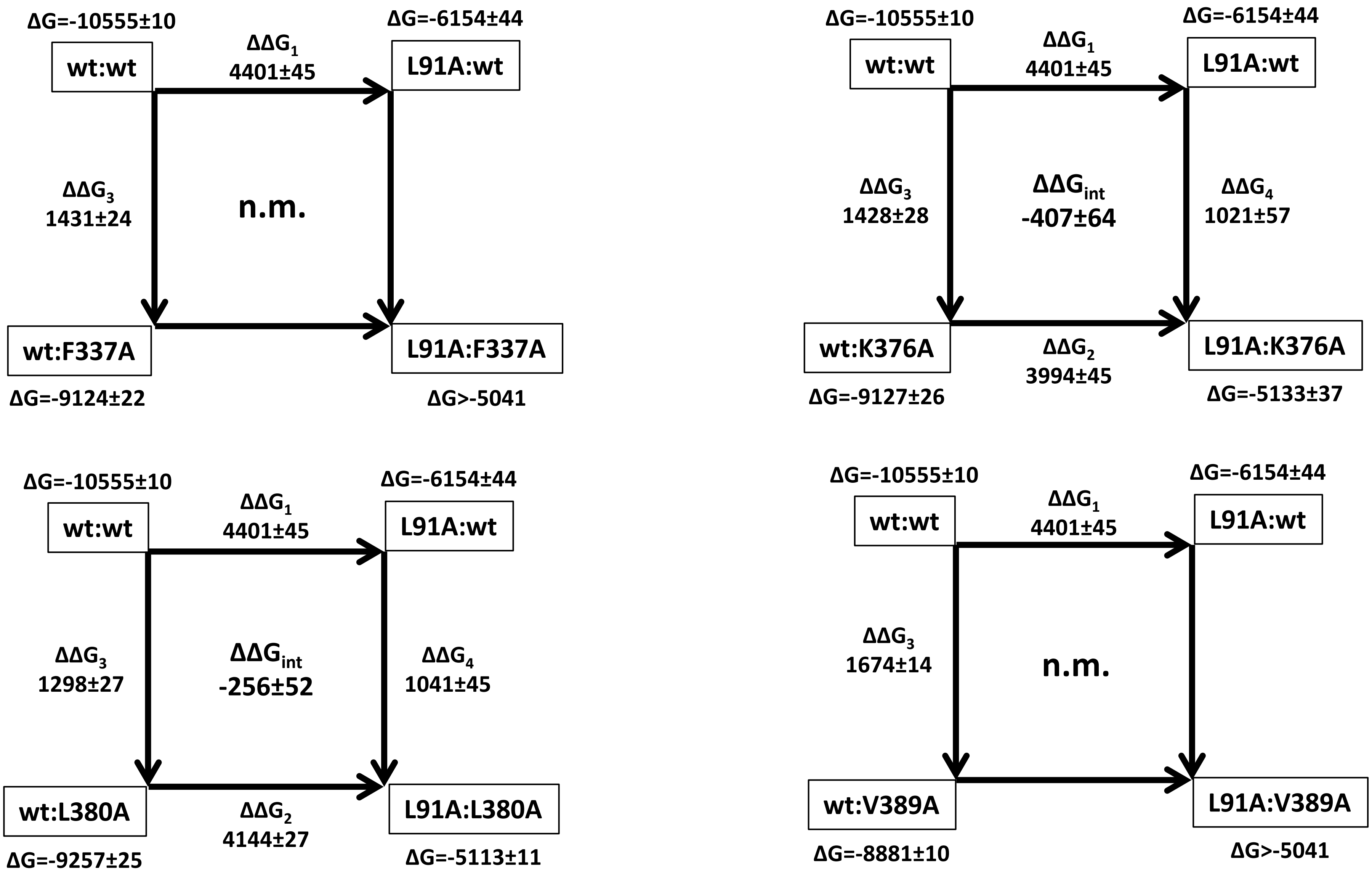

Figure 4-source data 1 – hYAP Leu91 – Ω-loop pocket

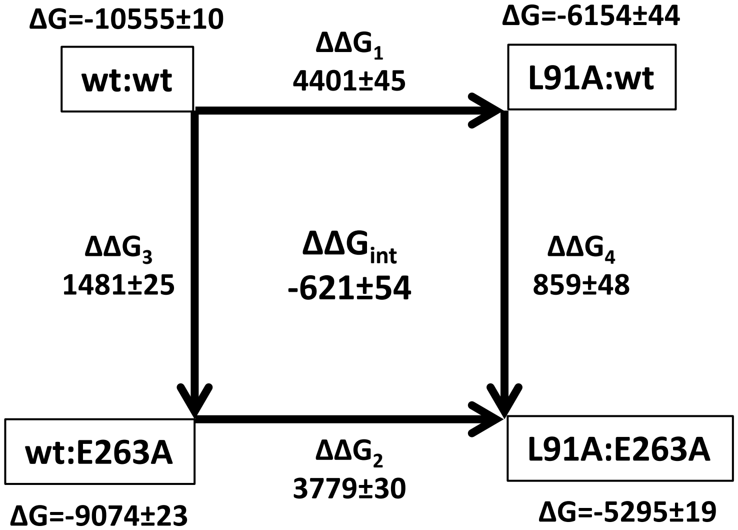

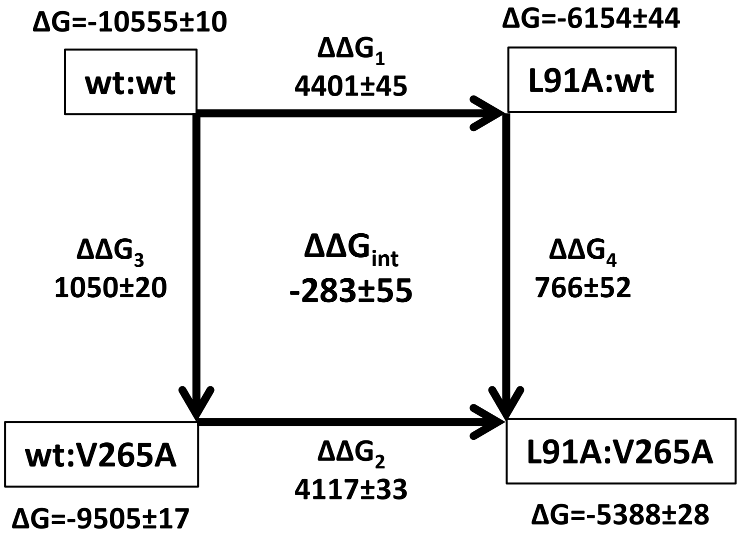

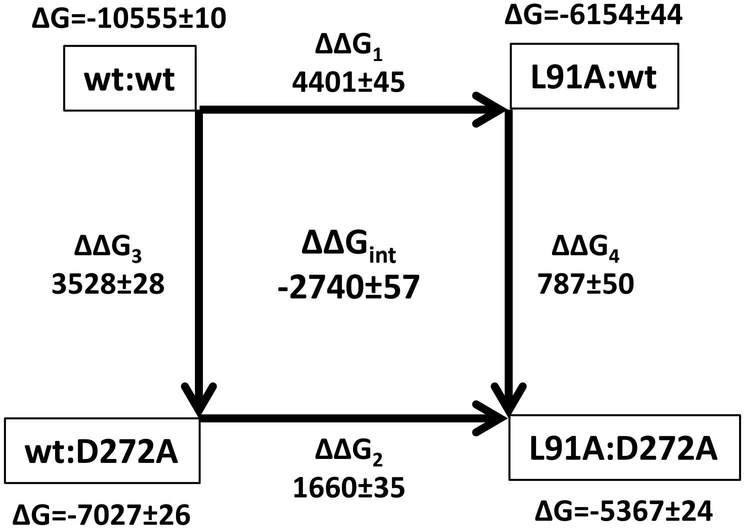

Figure 4-source data 1 – hYAP Ser94 – α-helix pocket

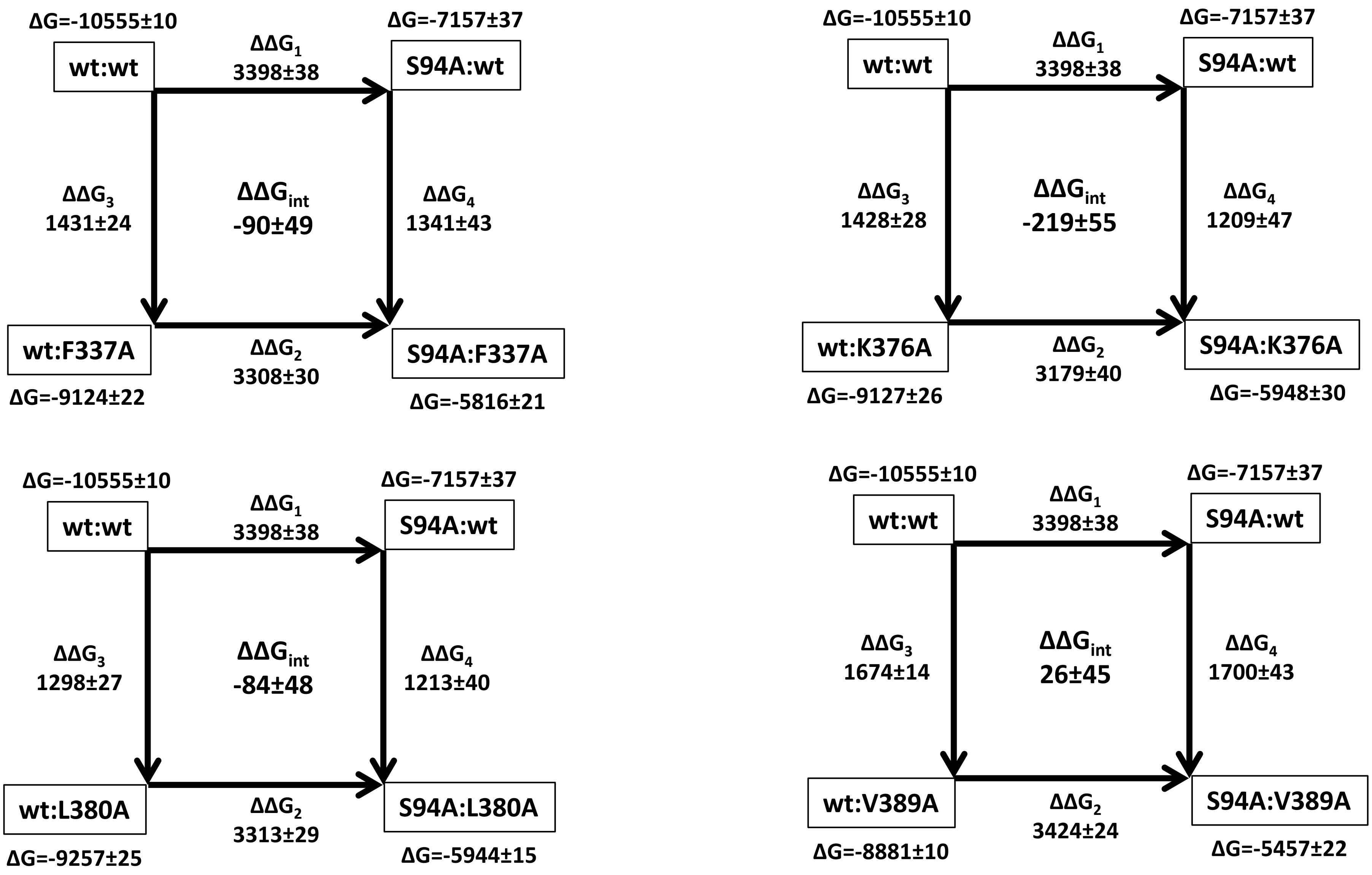

Figure 4-source data 1 – hYAP Ser94 – Ω-loop pocket

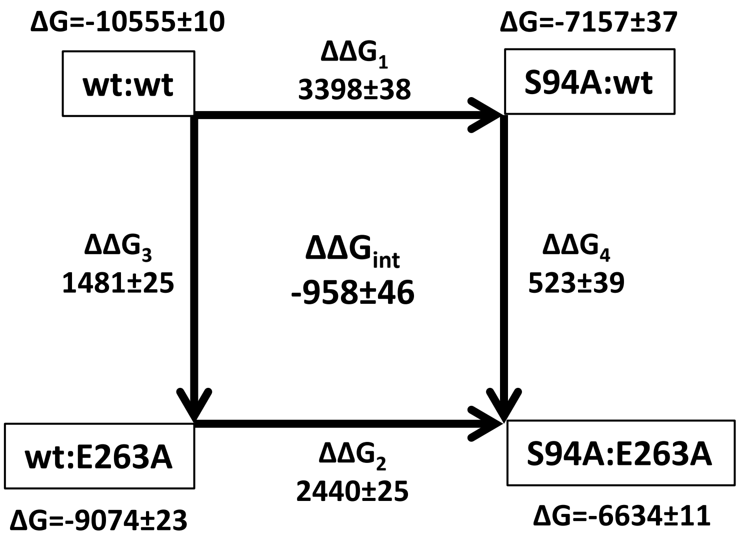

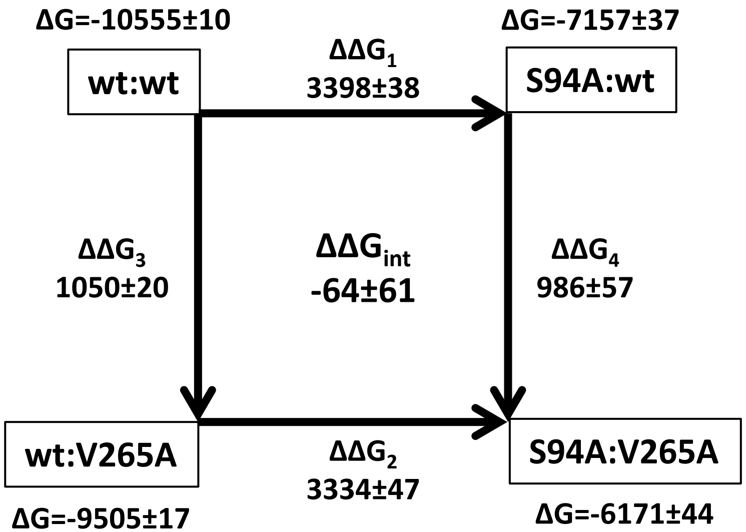

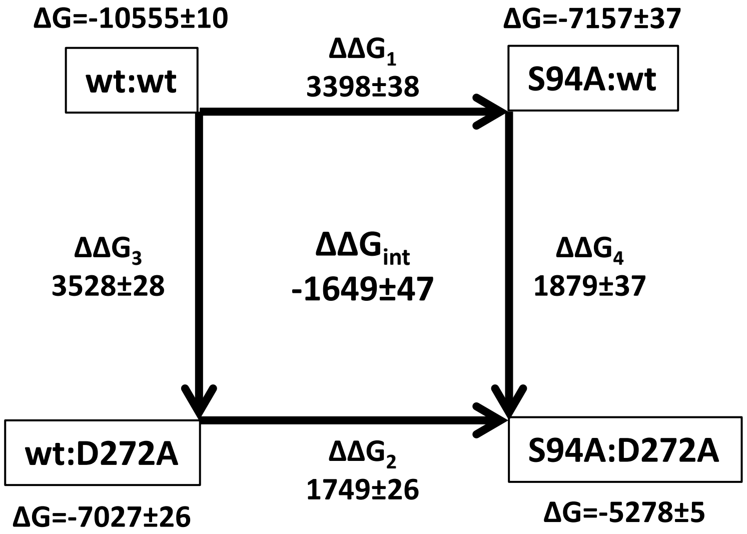

Figure 4-source data 1 – hYAP Phe95 – α-helix pocket

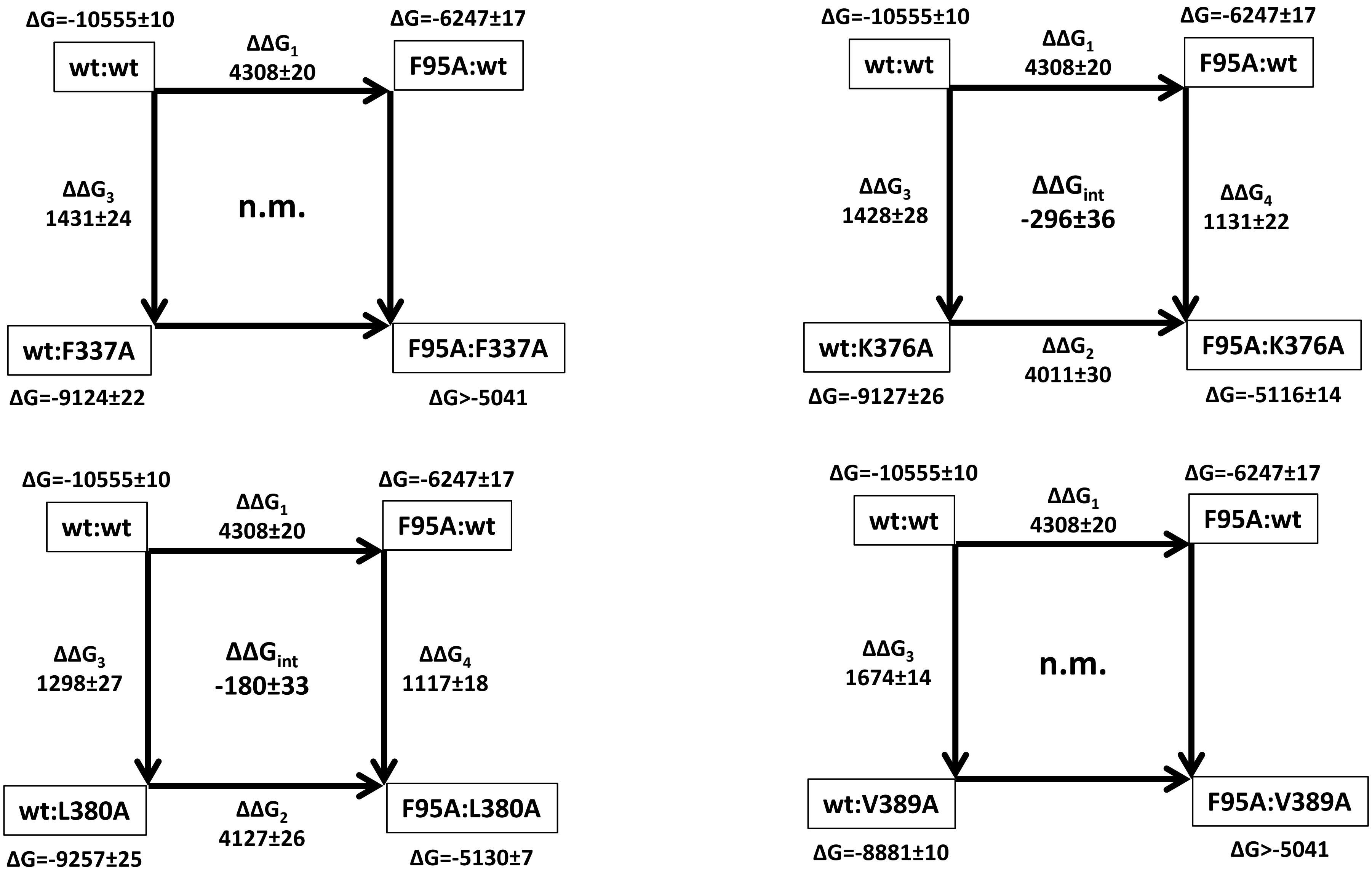

Figure 4-source data 1 – hYAP Phe95 – Ω-loop pocket

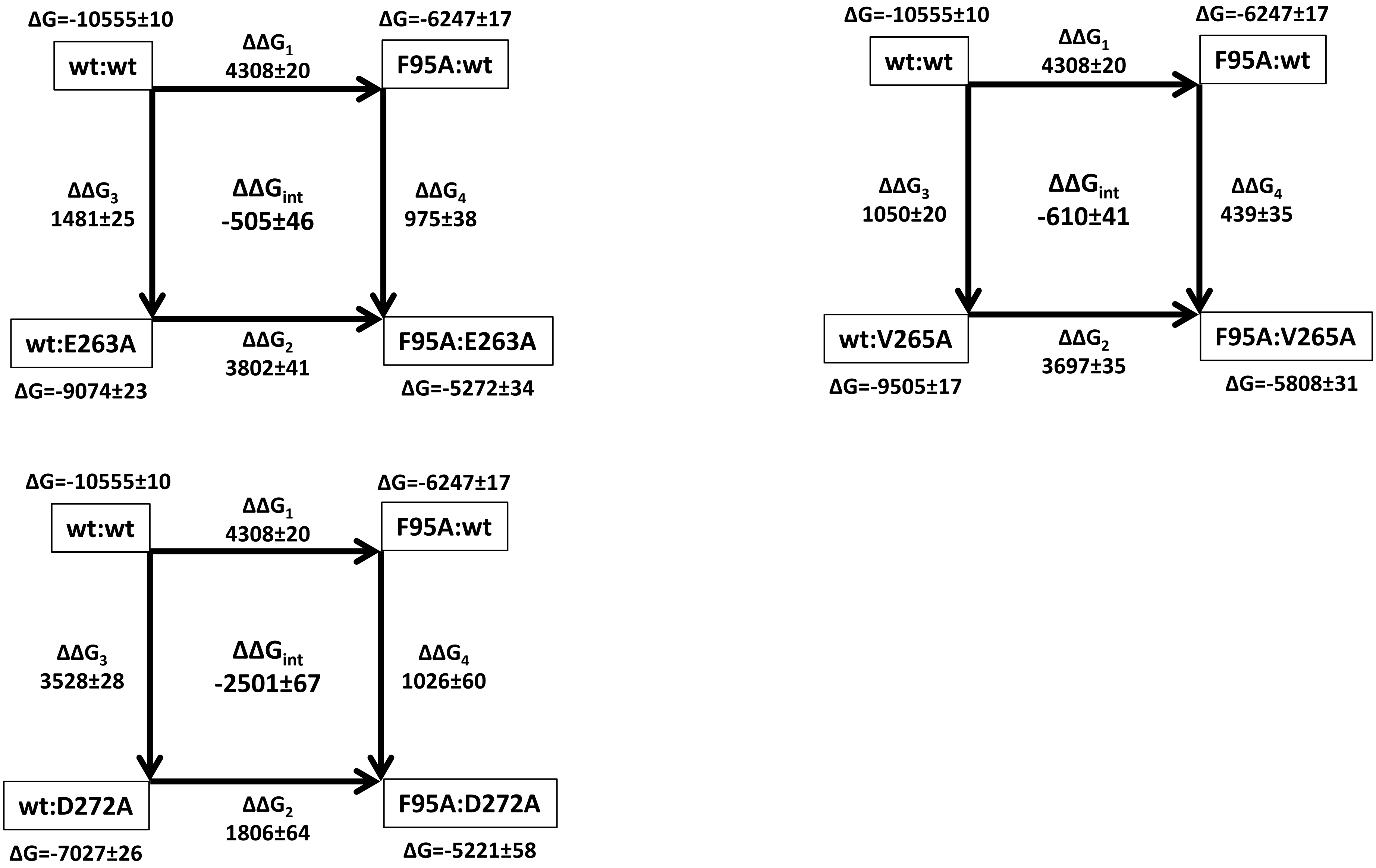

Figure 4-source data 1 – hYAP Phe96 – α-helix pocket

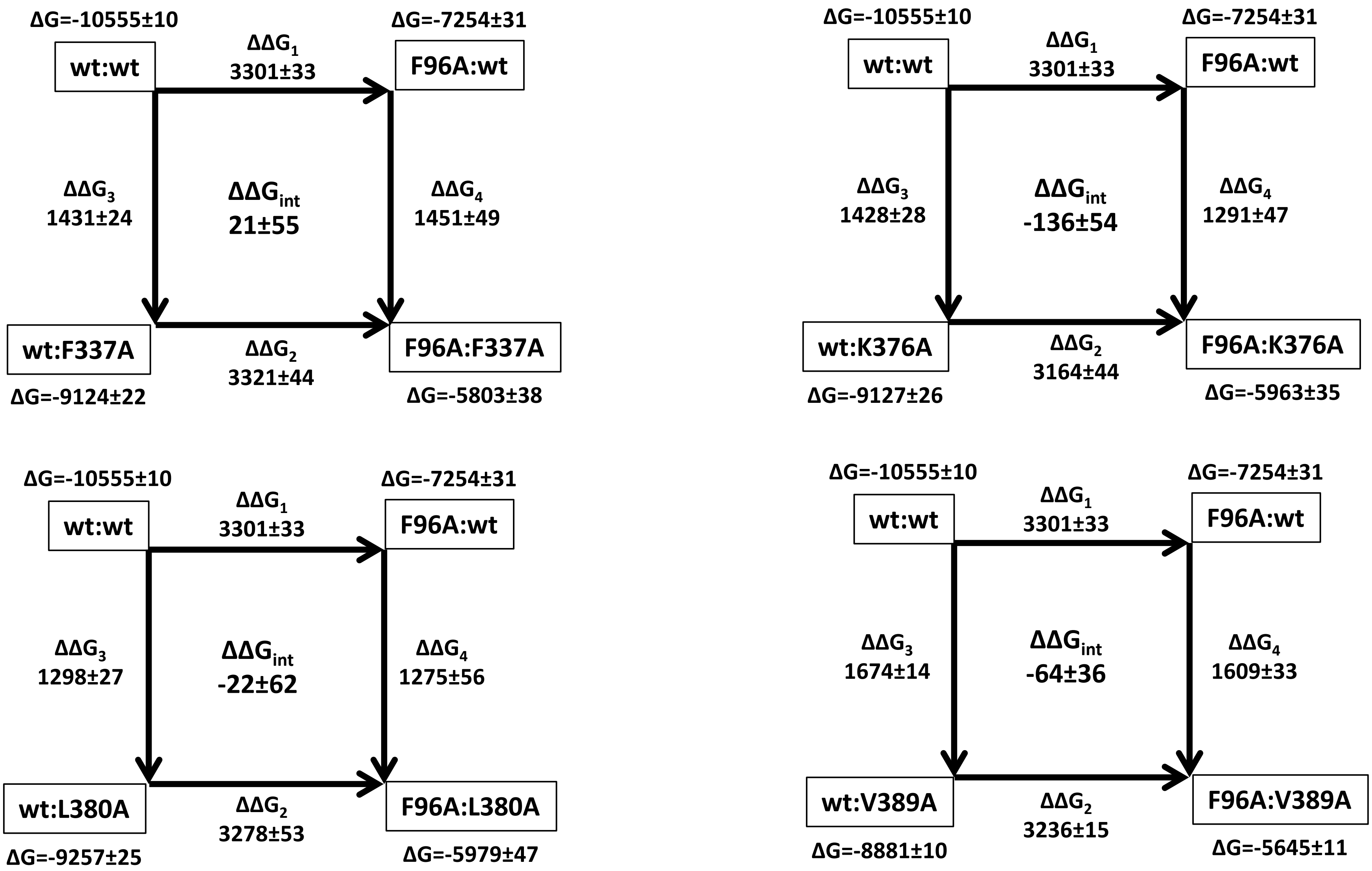

Figure 4-source data 1 – hYAP Phe96 – Ω-loop pocket

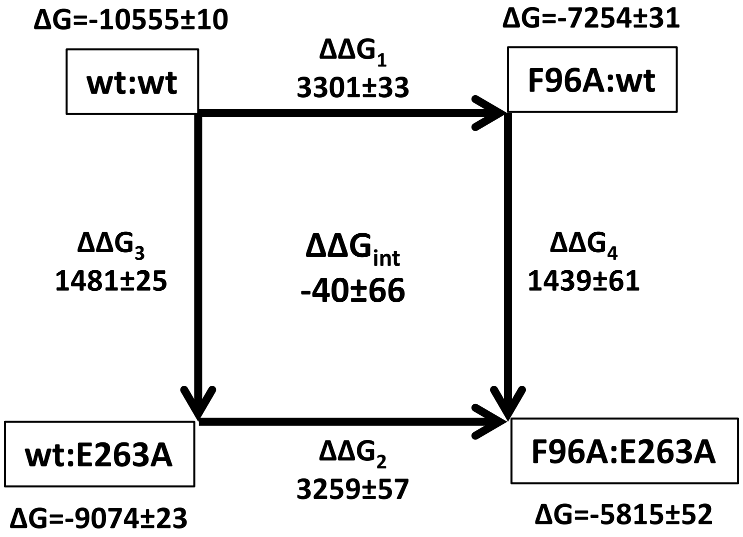

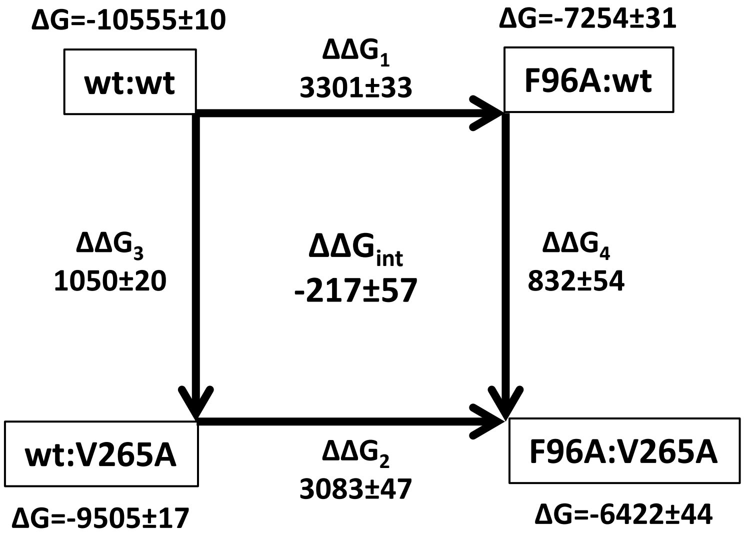

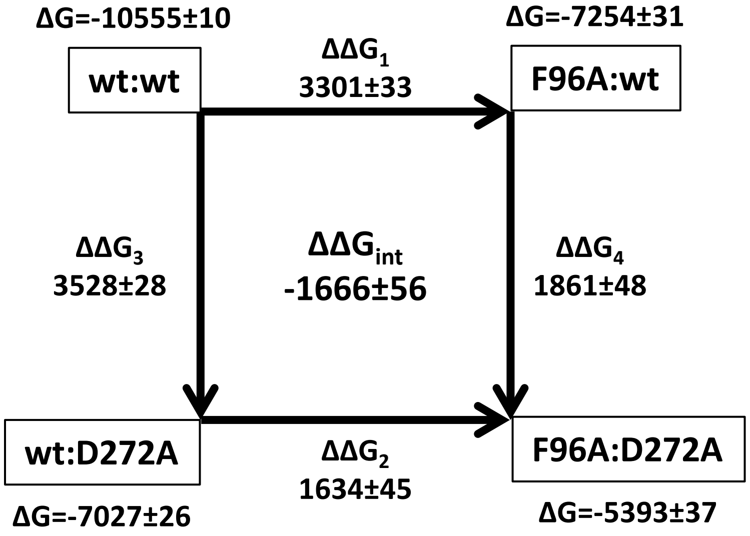

Supplement: Figure 4—source data 1. — The double mutant cycles have been grouped by hYAP residues. For explanations, see Figure 3 legend in the main text. The binding energies are in cal/mol and the standard errors (SE) are indicated. DOI: http://dx.doi.org/10.7554/eLife.25068.010 [file elife-25068-fig4-data1.pdf]
