# Supplementary material for: Dissection of the interaction between the intrinsically disordered YAP protein and the transcription factor TEAD
Source: eLife. 2017 Apr 21;6:e25068. doi: 10.7554/eLife.25068 (PMC5400505; doi:10.7554/eLife.25068)
Supplement: Supplementary file 1. — The N-Avitagged hTEAD4217-434 proteins were immobilized on sensor chips and their affinity for the hYAP50-171 proteins was measured at 298°K by Surface Plasmon Resonance in n ≥ 3 independent experiments. Kd values (in nM) were obtained from equilibrium data (Kdeq). Averages and standard errors (SE) are given. DOI: http://dx.doi.org/10.7554/eLife.25068.012 [file elife-25068-supp1.docx]

|  |  | |  | **hYAP^50-171^** | | | | | | | | |
| --- | --- | --- | --- | --- | --- | --- | --- | --- | --- | --- | --- | --- |
|  |  | |  | **α-helix** | | | **Ω-loop** | | | | | |
|  |  |  | wt | Leu65 | Leu68 | Phe69 | Met86 | Arg89 | Leu91 | Ser94 | Phe95 | Phe96 |
| **hTEAD^217-434^** |  | wt | 18  ±0 | 794  ±4 | 464  ±4 | 6447  ±400 | 2080  ±69 | 27423  ±381 | 30550  ±2250 | 5623  ±341 | 26045  ±755 | 4755  ±245 |
|  | **α-helix** | Phe337 | 202  ±8 | 4995  ±215 | 1745  ±105 | 14350  ±150 | 30825  ±1075 | 146500  ±500 | >  200000 | 54030  ±1900 | >  200000 | 55290  ±3560 |
|  |  | Lys376 | 203  ±9 | 2940  ±110 | 2587  ±155 | 8220  ±420 | 28725  ±1338 | 106936  ±2906 | 171667  ±10911 | 43287  ±2232 | 172094  ±3761 | 42247  ±2413 |
|  |  | Leu380 | 162  ±7 | 3863  ±248 | 2545  ±117 | 10605  ±736 | 20800  ±300 | 151667  ±4028 | 176950  ±3150 | 43500  ±1100 | 175133  ±1020 | 41200  ±3153 |
|  |  | Val389 | 304  ±5 | 5585  ±505 | 4873  ±365 | 16805  ±895 | 46113  ±1465 | >  200000 | >  200000 | 99020  ±3680 | >  200000 | 72070  ±1350 |
|  | **Ω-loop** | Glu263 | 220  ±9 | 8930  ±581 | 4838  ±63 | 65935  ±4515 | 28765  ±1935 | 123050  ±10050 | 130200  ±4200 | 13550  ±250 | 135500  ±7800 | 54300  ±4800 |
|  |  | Val265 | 106  ±3 | 7210  ±270 | 3520  ±85 | 46850  ±1970 | 14250  ±250 | 61140  ±1130 | 111533  ±5169 | 29710  ±2190 | 54840  ±2974 | 19450  ±1450 |
|  |  | Asp272 | 6995  ±317 | >  200000 | 173500  ±7100 | >  200000 | 35575  ±1306 | 29228  ±1684 | 115350  ±4750 | 133950  ±1050 | 148150  ±14550 | 110450  ±6950 |

**Supplementary file 1.**
