# Supplementary material for: Dissection of the interaction between the intrinsically disordered YAP protein and the transcription factor TEAD
Source: eLife. 2017 Apr 21;6:e25068. doi: 10.7554/eLife.25068 (PMC5400505; doi:10.7554/eLife.25068)
Supplement: Supplementary file 2. — Kd values were calculated (Kdcalc) assuming ΔΔGint = −500 cal/mol between the YAP:TEAD pairs. A calculation example for the hYAP Arg89:hTEAD4 Val389 pair is given in the following. The difference in binding energy between the two interactions wt hYAP:wt hTEAD4 and hYAP Arg89Ala:wt hTEAD4 is ΔΔG = 4338 cal/mol (Figure 4—source data 1, ΔΔG1 double mutant cycle for hYAP Arg89:hTEAD4 Val389). With ΔΔGint = −500 cal/mol, the difference in binding energy between the interactions wt hYAP:hTEAD4 Val389Ala and hYAP Arg89Ala:wt hTEAD4 Val389Ala should be ΔΔG = 3838 cal/mol. As ΔG = −8881 cal/mol for wt hYAP:hTEAD4 Val389Ala, the binding energy of the interaction between hYAP Arg89Ala and wt hTEAD4 Val389Ala should be ΔG = −5043 cal/mol. Using Kdcalc = eΔG/RT the dissociation constant for the interaction hYAP Arg89Ala:wt hTEAD4 Val389Ala should be Kdcalc = 199 µM. The last column gives the calculated Kd values for the interaction between the two mutant proteins (Kdadd) if no coupling exists between the two residues (ΔΔGint = 0 cal/mol in the above calculation). All Kdadd values are above 200 µM in agreement with our experimental data. DOI: http://dx.doi.org/10.7554/eLife.25068.013 [file elife-25068-supp2.docx]

| **hYAP mutant** | **hTEAD4 mutant** | **K_d_^calc^**  **(µM)** | **K_d_^add^**  **(µM)** |
| --- | --- | --- | --- |
| Arg89Ala | Val389Ala | 199 | 262 |
| Leu91Ala | Phe337Ala | 147 | 464 |
| Leu91Ala | Val389Ala | 222 | 342 |
| Phe95Ala | Phe337Ala | 126 | 516 |
| Phe95Ala | Val389Ala | 189 | 292 |
| Leu65Ala | Asp272Ala | 132 | 441 |
| Phe69Ala | Asp272Ala | 1070 | 308 |

**Supplementary file 2**.
